# A Low Ankle-Brachial Index and High Brachial-Ankle Pulse Wave Velocity Are Associated with Poor Cognitive Function in Patients Undergoing Hemodialysis

**DOI:** 10.1155/2019/9421352

**Published:** 2019-08-19

**Authors:** Ping-Hsun Wu, Yi-Ting Lin, Pei-Yu Wu, Jiun-Chi Huang, Szu-Chia Chen, Jer-Ming Chang, Hung-Chun Chen

**Affiliations:** ^1^Graduate Institute of Clinical Medicine, College of Medicine, Kaohsiung Medical University, Kaohsiung, Taiwan; ^2^Faculty of Medicine, College of Medicine, Kaohsiung Medical University, Kaohsiung, Taiwan; ^3^Division of Nephrology, Department of Internal Medicine, Kaohsiung Medical University Hospital, Kaohsiung Medical University, Kaohsiung, Taiwan; ^4^Department of Medical Sciences, Uppsala University, Uppsala, Sweden; ^5^Department of Family Medicine, Kaohsiung Medical University Hospital, Kaohsiung Medical University, Kaohsiung, Taiwan; ^6^Department of Internal Medicine, Kaohsiung Municipal Hsiao-Kang Hospital, Kaohsiung Medical University, Kaohsiung, Taiwan; ^7^Faculty of Renal Care, College of Medicine, Kaohsiung Medical University, Kaohsiung, Taiwan

## Abstract

Patients with end-stage renal disease (ESRD) have an increased risk of both impaired cognitive function and peripheral artery disease (PAD) than the general population. The association between PAD and dementia is recognized, but there are limited studies in patients with ESRD. The aim of this study was to evaluate the relationship between ankle-brachial index (ABI) and brachial-ankle pulse wave velocity (baPWV) and cognitive impairment in patients receiving hemodialysis (HD). We enrolled 136 prevalent HD patients (mean age 59.3 ± 10.5 years, 55.9% male). Cognitive performance was measured using the Montreal Cognitive Assessment (MoCA) and Cognitive Abilities Screening Instrument (CASI) by trained psychiatrists. Associations between the cognitive function and ABI and baPWV were assessed using multiple linear regression analysis. Compared with HD patients with ABI ≥ 0.9, patients with ABI < 0.9 had lower MoCA score (*p* = 0.027) and lower CASI score but did not achieve significant level (*p* = 0.056). In the multivariate stepwise linear regression analysis, ABI (per 0.1) was independently positively associated with the MoCA score (*β* coefficient = 0.62, *p* = 0.011) and the CASI score (*β* coefficient = 1.43, *p* = 0.026). There is a negative association between baPWV (per 100 cm/s) and CASI (*β* coefficient = −0.70, *p* = 0.009). In conclusion, a low ABI or high baPWV was associated with a lower cognitive function in HD patients.

## 1. Introduction

Patients with chronic kidney disease (CKD) or end-stage renal disease (ESRD) have a higher risk of dementia than the general population [1, 2], and patients with both dementia and ESRD have been associated with disability, hospitalization, dialysis withdrawal, and mortality [3–5]. As such, cognition should be evaluated in patients with ESRD to detect vascular and nonvascular dementia and prevent cognitive decline and its consequences. Given the high prevalence of dementia in patients with ESRD, identifying clinical markers that can predict cognitive dysfunction may be beneficial for both prevention and reducing health care costs. Scuteri et al. reported an association between arterial stiffness and brain injury and related brain pathologies [6]. They concluded that increased central pulse pressure and wave reflections may influence both the brain and kidneys and that it is likely that these phenomena are emphasized in ESRD patients.

Patients with ESRD have a high prevalence of peripheral artery disease (PAD), an important manifestation of systemic atherosclerosis [7]. PAD shares similar risk factors with coronary artery disease and cerebrovascular disease [8]. The ankle-brachial index (ABI) and pulse wave velocity (PWV) are common noninvasive tools used to quantitatively assess arterial health with regard to blocked arteries and arterial stiffness, respectively. PAD has been associated with an increased risk of both cardiovascular disease [9] and cognitive dysfunction in the general population [10], and a low ABI has been reported to predict the future risk of cognitive impairment [11] and dementia [12].

However, few studies have evaluated the association between PAD and cognitive function in patients with CKD or ESRD [13, 14], despite being at high risk of atherosclerosis and dementia. Moreover, no previous study has simultaneously evaluated ABI and PWV as markers of cognitive function in ESRD patients. A better understanding of the relationship between PAD and cognitive decline in ESRD patients may provide new insights into the physiological mechanisms of the prodromal stage of dementia. Accordingly, the aim of this study was to assess the association between ABI and brachial-ankle PWV (baPWV) and cognitive function in patients receiving hemodialysis (HD).

## 2. Methods

### 2.1. Study Subjects and Design

This study was conducted at a dialysis clinic in a regional hospital in southern Taiwan from August 2016 to January 2017. All patients received regular HD three times per week with high-flux dialyzers and a blood flow rate of 250-300 mL/min, dialysate flow rate of 500 mL/min, with each HD session lasting for 3.5-4 hours. Patients aged > 30 years who had received maintenance dialysis for at least 90 days were recruited. After excluding the patients who refused to undergo ABI-form device (*n* = 5) or neuropsychological (*n* = 17) examinations, patients with atrial fibrillation (*n* = 4), patients with bilateral below-the-knee amputations (*n* = 3), and patients who had been hospitalized for 4 weeks prior to study enrollment (*n* = 5), the remaining 136 patients (76 men and 60 women) were included into the final analysis. The study protocol was approved by the Institutional Review Board of Kaohsiung Medical University Hospital (KMUHIRB-E(I)-20160095), and written informed consent was obtained from all patients. All clinical investigations were conducted according to the principles expressed in the Declaration of Helsinki.

### 2.2. Demographic, Medical, and Laboratory Data

Demographic and medical data including sex, age, smoking history (ever versus never), and comorbidities were obtained from interviews and the patients' medical records. Body mass index was calculated as weight divided by height squared in kg/m^2^. Hypertension was defined as blood pressure ≥ 140/90 mmHg or taking antihypertensive drugs, and diabetes was defined as fasting blood glucose level of ≥126 mg/dL or taking antidiabetic drugs. Patients with a history of cerebrovascular accidents, including cerebral bleeding and infarction, were defined as having a cerebrovascular disease, and those with a history of angina, ischemic changes in electrocardiography, old myocardial infarction, or coronary bypass surgery/angioplasty were defined as having coronary artery disease. Laboratory examinations were performed in fasting blood samples obtained ≤1 month of enrollment using an autoanalyzer (COBAS Integra 400, Roche Diagnostics GmbH, Mannheim, Germany).

### 2.3. Measurement of ABI and baPWV

Because ABI and baPWV might be influenced by hemodialysis [15], all the values of ABI and baPWV were measured 10–30 minutes before hemodialysis. The values of ABI and baPWV were measured by using an ABI-form device (VP1000; Colin Co. Ltd., Komaki, Japan), which automatically and simultaneously measures blood pressures in both arms and ankles using an oscillometric method [16]. Occlusion and monitoring cuffs were placed tightly around the upper arms without blood access and both sides of the lower extremities in the supine position. ABI was calculated by the ratio of the ankle systolic blood pressure divided by the arm systolic blood pressure, and the lower value of the ankle systolic blood pressure was used for the calculation. For measuring baPWV, pulse waves obtained from the brachial and tibial arteries were recorded simultaneously, and the transmission time, which was defined as the time interval between the initial increase in brachial and tibial waveforms, was determined. The transmission distance from the arm to each ankle was calculated according to body height. The baPWV value was automatically computed as the transmission distance divided by the transmission time. After obtaining bilateral baPWV values, the highest one was used as a representative for each subject. The ABI and baPWV measurements were done once in each patient. Concerning the reproducibility of ABI and baPWV, we randomly evaluated 25 patients at least 15 minutes apart for the reproducibility of ABI and baPWV by using 2 separate measurements. Mean percent error was calculated as the absolute difference divided by the average of the 2 observations.

### 2.4. Cognitive Function Assessment

Three cognitive function tests evaluated in this study were Montreal Cognitive Assessment (MoCA) [17, 18] and Cognitive Abilities Screening Instrument (CASI) [18, 19] (Supplementary [Supplementary-material supplementary-material-1]). The MoCA is a more sensitive 30-point screening tool that includes copying a cube, verbal abstraction, serial subtraction, drawing a clock, a 5-word learning and delayed recall task, naming an animal, digit span backward and forwards, selective attention, repeating a sentence, phonemic word fluency, and spatial and temporal orientation. These tasks encompass multiple domains of cognition, including short-term memory, visuospatial ability, and executive function, language, attention, concentration and working memory, and orientation to time and place. The CASI assesses a broad range of cognitive domains using a 40-item global cognitive test, which contains nine cognitive evaluation domains, including long-term memory, short-term memory, orientation, attention, mental manipulation, list-generating fluency, language, abstraction/judgment, and drawing (Supplementary [Supplementary-material supplementary-material-1]).

### 2.5. Statistical Analysis

Descriptive statistics were presented as percentages, means ± standard deviations, or medians (25^th^-75^th^ percentile) for HD duration and triglycerides. Differences between groups were analyzed using the chi-square test for categorical variables and the independent *t*-test for continuous variables with approximately normal distribution or the Mann-Whitney *U* test for continuous variables with skewed distribution. Multiple stepwise linear regression analyses were used to identify the factors associated with cognitive function (MoCA and CASI). The adjusted multivariate variables in the models included age, sex, smoking, a history of hypertension, diabetes, cerebrovascular and coronary artery diseases, systolic and diastolic blood pressures, body mass index, log-transformed hemodialysis duration, cause of end-stage renal disease, albumin, log-transformed triglyceride, total cholesterol, hemoglobin, creatinine, calcium-phosphorus product, Kt/V, and amount of ultrafiltration. Relevant demographic parameters were also analyzed by a backward stepwise selection with *p* values for independent variables to enter and to stay in the models set at 0.1 and subsequently a final elimination step at *p* < 0.05. All statistical analyses were performed using SPSS version 19.0 for Windows (SPSS Inc., Chicago, IL, USA) and STATA version 14 (StataCorp LP, College Station, TX, USA). A two-tailed *p* value of <0.05 was considered to be statistically significant.

## 3. Results

### 3.1. Demographic and Clinical Characteristics

The mean age of the 136 HD patients was 59.3 ± 10.5 years, 55.9% were men, and 21.3% had an ABI < 0.9. The mean percent error for ABI and baPWV measurement was 3.58 ± 3.15% and 5.8% ± 5.0%, respectively. The patients were stratified into two groups according to ABI < 0.9 or ≥0.9. Comparisons of the clinical characteristics between the two groups are shown in Table 1. Compared to the ABI ≥ 0.9 groups, the ABI < 0.9 groups were older and had more diabetes-related comorbidities, lower systolic and diastolic blood pressures, and higher levels of calcium-phosphorus products. Regarding cognitive function, compared to the ABI ≥ 0.9 groups, the ABI < 0.9 groups had lower MoCA (*p* = 0.027) scores. The ABI < 0.9 group also had a lower CASI score, but the difference was not significant (*p* = 0.056).

### 3.2. Associations of ABI and Cognitive Function in HD Patients

In univariate linear regression models, ABI was positively associated with MoCA (*β* coefficient 1.07, 95% confidence interval (CI) 0.55 to 1.59, and *p* < 0.001) and CASI (*β* coefficient 2.87, 95% CI 1.45 to 4.29, and *p* < 0.001) (Table 2). In multivariate linear regression models with stepwise backward covariate selection, ABI was persisted positively associated with MoCA (*β* coefficient 0.62, 95% CI 0.14 to 1.09, and *p* = 0.011) and CASI (*β* coefficient 1.43, 95% CI 0.17 to 2.70, and *p* = 0.026) (Table 2). The stepwise backward covariate selection models of ABI and cognitive function (MoCA and CASI) were demonstrated in Supplementary Tables [Supplementary-material supplementary-material-1]-[Supplementary-material supplementary-material-1]. Since extremely high ABI (>1.3) is correlated to multiple comorbidities or arterial calcification, we reanalyzed the association between ABI and cognitive function test after excluding subjects with ABI value > 1.3. Similar results found the positive association between ABI and the MoCA or CASI score in univariate linear regression analysis (*β* coefficient 1.08, 95% CI 0.55 to 1.61, and *p* < 0.001 in MoCA; *β* coefficient 2.97, 95% CI 1.53 to 4.42, and *p* < 0.001 in CASI) and multivariate stepwise linear regression analysis (*β* coefficient 0.63, 95% CI 0.15 to 1.11, and *p* = 0.011 in MoCA; *β* coefficient 1.36, 95% CI 0.04 to 2.69, and *p* = 0.043 in CASI).

### 3.3. Associations of baPWV and Cognitive Function in HD Patients

In univariate linear regression models, baPWV was negatively associated with MoCA (*β* coefficient -0.33, 95% CI -0.56 to -0.11, and *p* = 0.004) and CASI (*β* coefficient -1.16, 95% CI -1.76 to -0.56, and *p* < 0.001) (Table 3). In multivariate linear regression models with stepwise backward covariate selection, baPWV was persisted negatively associated with CASI (*β* coefficient -0.70, 95% CI -1.22 to -0.18, and *p* = 0.009) but not with MoCA (*β* coefficient -0.075, 95% CI -0.31 to 0.16, and *p* = 0.52) (Table 3). The stepwise backward covariate selection models of baPWV and cognitive function (MoCA and CASI) were demonstrated in Supplementary Tables [Supplementary-material supplementary-material-1]-[Supplementary-material supplementary-material-1]. Since both sides of PAD could be found in patients with HD that influence the baPWV data, we reanalyzed the association between baPWV and cognitive function test after excluding subjects with ABI value < 0.9. Similar results found the negative association between baPWV and MoCA (*β* coefficient -0.38, 95% CI -0.63 to -0.12, and *p* = 0.004) or CASI score (*β* coefficient -1.01, 95% CI -1.65 to -0.37, and *p* = 0.002) in the univariate linear regression analysis. However, the insignificant negative association was demonstrated in stepwise linear regression approach (*β* coefficient -0.20, 95% CI -0.45 to 0.06, and *p* = 0.13 in MoCA; *β* coefficient -0.44, 95% CI -1.07 to 0.18, and *p* = 0.16 in CASI).

### 3.4. Subgroup Analysis of ABI or baPWV and Cognitive Function in HD Patients

**Figure 1:**
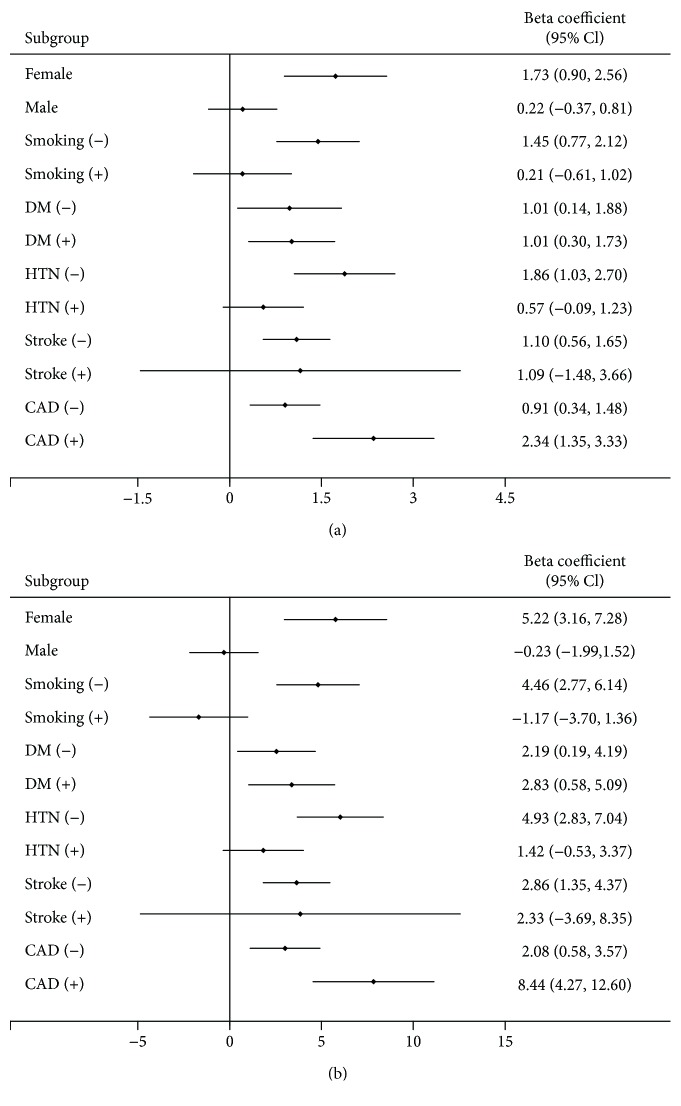
Subgroup analysis of the association between ABI and cognitive function test; (a) MoCA test and (b) CASI test. DM: diabetes mellitus; HTN: hypertension; CAD: coronary artery disease.

**Figure 2:**
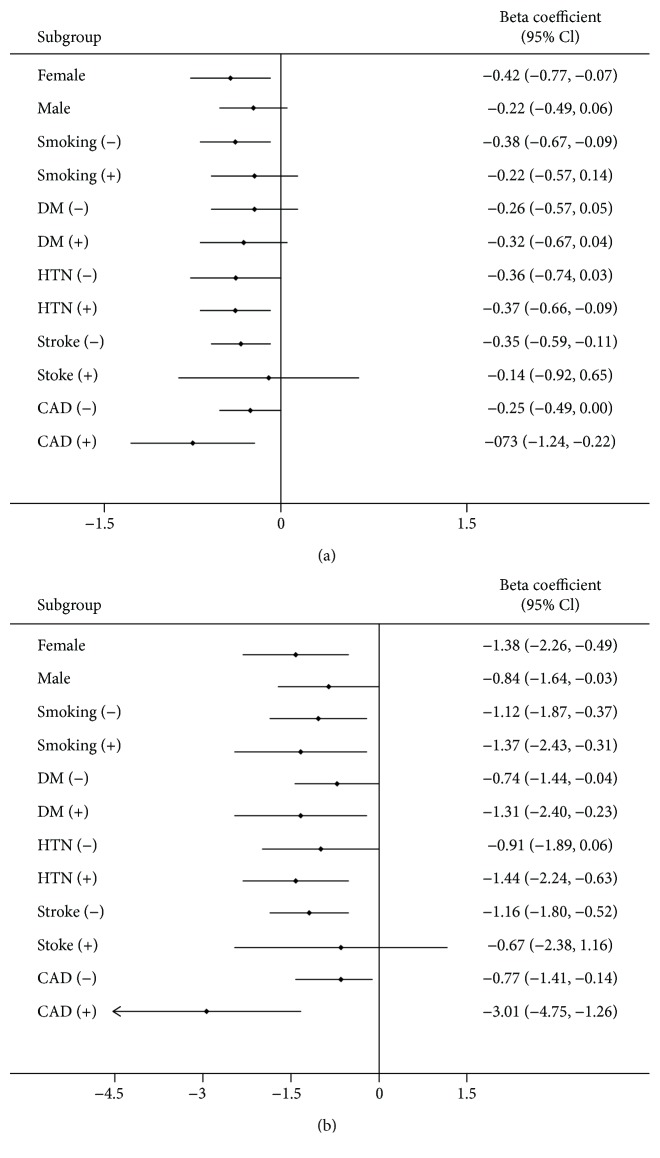
Subgroup analysis of the association between baPWV and cognitive function test; (a) MoCA test and (b) CASI test. DM: diabetes mellitus; HTN: hypertension; CAD: coronary artery disease.

Subgroup analysis of gender and baseline comorbidities demonstrated that ABI was positively associated with cognitive function except for the male gender, smoking habit, and patients with hypertension and stroke comorbidities (Figure 1). The baPWV was negatively associated with cognitive function except for male, smoking habit, patients with/without diabetes comorbidity, no hypertension comorbidity, or those with a stroke history in MoCA. A similar pattern was found in the CASI test (Figure 2).

### 3.5. Sensitivity Analysis of ABI or baPWV and Cognitive Function in HD Patients

We evaluated the comorbidities and cognitive function test (MoCA and CASI) association. Only diabetes was negatively associated with cognitive function test score (Supplementary [Supplementary-material supplementary-material-1]). Considering diabetes as an important confounder for cognitive function, sensitivity analysis of stepwise regression models with additional diabetes comorbidity adjustment in ABI or baPWV and cognitive function was analyzed. A similar result was found (Supplementary Tables [Supplementary-material supplementary-material-1] and [Supplementary-material supplementary-material-1]).

## 4. Discussion

This study examined the relationship between PAD and cognitive performance in 136 prevalent HD patients. PAD was assessed according to ABI and baPWV, and cognitive performance was assessed according to MoCA and CASI, which collectively evaluated memory, orientation, attention, visual screening, motor speed, planning abilities, executive function, and language. A low ABI was associated with low MoCA and CASI scores, and a high baPWV was associated with low CASI scores. Since cognition was evaluated using questionnaires with different sensitivities and specificities, it would be promising results to find the association between ABI or baPWV and cognition in HD patients.

The prevalence of cognitive impairment is high in patients undergoing HD [20–22]. Considering the different definition of cognitive impairment in patients with kidney disease based on sensitivity and specificity, MoCA is a valid, more sensitive, and well-suited screening tool for cognitive impairment in patients receiving HD [23, 24]. The optimal cut-off of ≤24 points out of a 30-point maximum is lower than the cut-off value of ≤26 described in the original data collected in a population of patients with Alzheimer's disease and mild cognitive impairment [25]. In the present HD cohort, there is a higher prevalence of cognitive impairment (84.6%; 114/136 based on MoCA cut‐off ≤ 24 points) than other HD cohorts [26]. This may be related to longer dialysis vintage and higher diabetes mellitus comorbidity in the HD cohort.

The first important finding of this study is that a low ABI was associated with poor cognitive function. The etiology for cognitive impairment in HD patients is complex, including traditional risk factors (old age, hypertension, diabetes, and smoking), nontraditional risk factors (anemia, albuminuria, inflammation, homocysteinemia, and malnutrition), and dialysis-related factors (hypotension, all of which can lead to chronic cerebral hypoxemia or edema) [27, 28]. In addition, high rates of small and large cerebral vascular injuries, including white matter lesions, subcortical atrophy, lacunar infarcts, and microbleeds, have been reported in patients with CKD in brain imaging studies [29, 30]. Vascular stiffness and abnormalities in structure and function have also been associated with cognitive decline and stroke [31]. The ABI, an easily obtained good marker of atherosclerosis [32, 33], has been shown to be a good prognostic marker of atherosclerosis for stroke. Subjects with a low ABI have been reported to have more atherosclerotic and vascular conditions, and these conditions may lead to generalized endothelial dysfunction [34], stenosis of intracranial and extracranial arteries [35], and decreased cerebral perfusion inducing oxidative stress [36]. Taken together, these findings may explain the association between low ABI and poor overall cognitive function [37]. A low ABI has also been correlated with global cortical thinning and reduced cortical thickness in the limbic, parietal, temporal, and occipital lobes [38]. A decrease in overall cerebral perfusion due to atherosclerosis may cause pathological changes such as infarcts or neurodegeneration [39]. The present study declared that a low ABI was associated with the risk of poor cognitive function in HD patients, as indicated by low MoCA and CASI scores.

The second important finding of this study is that a high baPWV was associated with poor cognitive function. Arterial stiffness is a marker of functional and structural changes in the arteries and can contribute to microvascular brain diseases, and it can be assessed noninvasively using PWV measurements. Several studies have examined the association between arterial stiffness and cognitive function in the general population [40, 41] and in patients with CKD or ESRD [13, 14]. Longitudinal studies have indicated that high values of PWV can predict lower cognitive function score in the elderly [42]. One study with 72 ESRD patients undergoing HD showed that a higher carotid-femoral PWV was associated with cognitive impairment [14]. Another study suggests an inverse association between baPWV and cognitive function among the elderly [43]. The present study demonstrated a negative association between baPWV and cognitive function score (CASI). Thus, baPWV could be considered to be a marker of cognition in ESRD patients.

In the present study, diabetes is significantly negatively associated with the cognitive function test score. However, previous cerebrovascular event history is not significantly negatively associated with the cognitive function test score because of a few patients with old stroke hospitalization records in our study. Thus, sample sizes are insufficient to found the association. Therefore, the main results were analyzed by multivariate stepwise variable selection model in this study. Stepwise regression is a method of fitting regression models in which the choice of predictive variables is carried out by an automatic procedure. At each step, a variable is considered for addition to or subtraction from the set of explanatory variables based on some prespecified criterion. Since diabetes is an important confounder for cognitive function, we further sensitivity analyzed the stepwise regression models with additional diabetes comorbidity adjustment, the results remain similar. In our subgroup analysis, the association between ABI/baPWV and cognitive function was still significant in diabetes mellitus strata. Thus, the relationship between ABI/baPWV and cognition was similar in patients with and without diabetes comorbidity.

Besides, a positive association was found between the duration of dialysis and cognitive function test (CASI) in the multivariate linear regression model. Although this association is counterintuitive, it could reflect a selection bias or survivor bias inherent in prevalent dialysis cohorts. Survivors of long-term dialysis often begin dialysis at a young age and have had no opportunity for renal transplantation. Young and healthy dialysis patients would actually have better cognitive function than older or more ill dialysis patients. In the stepwise selection model, the significant association between the duration of dialysis and the cognitive function test may be related to open a noncausal path because of nonconfounder (Collider variables) adjustment in the causal pathway between duration of dialysis and cognitive function. On the contrary, our study interest is the association between ABI/baPWV and cognitive function that variable selection fits the causal pathway models in our study.

The important clinical implications of our findings are that ABI and baPWV are noninvasive and easily measured parameters that can be used to help identify the HD patients at risk of cognitive dysfunction by evaluating the degree of arterial stiffness. However, there are several limitations to this study. First, a causal relationship could not be inferred due to the cross-sectional nature of the study. Nonetheless, our results may help to identify the risk of cognitive dysfunction in HD patients through regular ABI examinations. Since a long-term decline in cognitive function could not be confirmed in this study, future prospective studies are needed to address this issue. Second, this study was conducted at a single regional hospital, thereby limiting the selection and the number of patients. In addition, patients with peritoneal dialysis were not enrolled in this study; therefore, the results may not be applicable to patients undergoing peritoneal dialysis. Further studies are needed to confirm our findings. Third, ABI measurements and cognitive function tests were performed only once in each patient, which may have caused misclassification. Fourth, not all possible parameters were included in this study such as dietary habits, genetic factors, and medications. In addition, neuroimaging assessments could not be performed, so asymptomatic brain lesions could not be fully investigated. Moreover, different types of dementia and cognitive impairment etiology may have affected the results.

## 5. Conclusion

In this study, the HD patients with a low ABI and high baPWV were associated with a lower cognitive function, even after adjusting for age and hemodynamic and risk factors for atherosclerosis. Future studies should explore the longitudinal associations between ABI and baPWV and cognitive decline in a representative sample of HD patients. Our findings should serve to remind physicians of the importance of evaluating PAD in addition to cognitive function in HD patients.

## Figures and Tables

**Table 1:** Comparison of baseline characteristics in patients categorized by ABI < 0.9 or ≥0.9.

Characteristics	All patients (*n* = 136)	ABI ≥ 0.9 (*n* = 107)	ABI < 0.9 (*n* = 29)	*p*
Age	59.3 ± 10.5	58.1 ± 9.8	63.7 ± 12.1	0.011
Male gender (%)	55.9	57.9	48.3	0.352
Smoking history (%)	37.3	37.4	37.0	0.973
Diabetes mellitus (%)	42.5	36.4	66.7	0.005
Hypertension (%)	56.7	55.1	63.0	0.463
Coronary artery disease (%)	7.5	6.5	11.1	0.419
Cerebrovascular disease (%)	6.0	4.7	11.1	0.207
Systolic blood pressure (mmHg)	156.2 ± 24.5	158.8 ± 23.0	146.5 ± 27.9	0.016
Diastolic blood pressure (mmHg)	82.0 ± 14.5	84.8 ± 13.5	71.5 ± 13.3	<0.001
Body mass index (kg/m^2^)	24.0 ± 3.9	23.9 ± 4.0	24.2 ± 3.2	0.734
ABI	0.98 ± 0.19	1.06 ± 0.10	0.69 ± 0.14	<0.001
baPWV (cm/s)	1870.4 ± 441.4	1897.5 ± 389.1	1768.7 ± 593.7	0.164
Duration of hemodialysis (years)	7.5 (3.1-13.0)	7.8 (3.1-13.0)	5.9 (3.1-12.5)	0.650
Cause of end-stage renal disease				0.289
Hypertension (%)	5.9	5.6	6.9	
Diabetes mellitus (%)	40.4	36.4	55.2	
Glomerulonephritis (%)	49.3	53.3	34.5	
Others^∗^ (%)	4.4	4.7	3.4	
Laboratory parameters				
Albumin (g/dL)	3.9 ± 0.3	3.9 ± 0.3	3.8 ± 0.3	0.216
Triglyceride (mg/dL)	124.5 (83-195.5)	120.5 (82.75-189.5)	138.5 (83.25-234.25)	0.277
Total cholesterol (mg/dL)	173.6 ± 40.6	172.8 ± 39.6	176.8 ± 44.7	0.638
Hemoglobin (g/dL)	10.5 ± 1.4	10.5 ± 1.4	10.8 ± 1.4	0.252
Creatinine (mg/dL)	10.1 ± 2.1	10.1 ± 2.1	9.9 ± 2.2	0.606
CaXP product (mg^2^/dL^2^)	45.5 ± 11.8	44.4 ± 11.1	49.6 ± 13.8	0.038
Dialysis dose (Kt/V)	1.62 ± 0.26	1.62 ± 0.26	1.60 ± 0.28	0.775
Total net fluid loss (amount of ultrafiltration) (kg)	2.70 ± 1.08	2.74 ± 1.04	2.53 ± 1.26	0.356
Cognitive function assessment				
MoCA (score)	18.7 ± 6.0	19.5 ± 5.3	16.0 ± 7.5	0.027
CASI (score)	75.6 ± 16.4	77.5 ± 13.5	68.5 ± 23.3	0.056

Abbreviation: ABI: ankle-brachial index; baPWV: brachial-ankle pulse wave velocity; CaXP product: calcium × phosphorus product; MoCA: Montreal Cognitive Assessment; CASI: Cognitive Abilities Screening Instrument. ^∗^Other causes of end-stage renal disease include polycystic kidney disease, tumor, systemic lupus erythematosus, gout, and interstitial nephritis.

**Table 2:** The association between ABI and cognitive function test (MoCA and CASI) using univariate linear regression analysis and multivariate stepwise linear regression analysis.

Cognitive function test	Univariate		Multivariate (stepwise)^∗^	
	*β* coefficient (95% CI)	*p* value	*β* coefficient (95% CI)	*p* value
MoCA	1.07 (0.55 to 1.59)	<0.001	0.62 (0.14 to 1.09)	0.011
CASI	2.87 (1.45 to 4.29)	<0.001	1.43 (0.17 to 2.70)	0.026

^∗^Adjusting for stepwise procedure selected covariates (age, sex, smoking habit, a history of diabetes, hypertension, coronary artery disease, and cerebrovascular disease, systolic and diastolic blood pressures, body mass index, log-transformed hemodialysis duration, cause of end-stage renal disease, albumin, log-transformed triglyceride, total cholesterol, hemoglobin, creatinine and calcium-phosphorus product, Kt/V, and amount of ultrafiltration).

**Table 3:** The association between baPWV and cognitive function test (MoCA and CASI) using univariate linear regression analysis and multivariate stepwise linear regression analysis.

Cognitive function test	Univariate		Multivariate (stepwise)^∗^	
	*β* coefficient (95% CI)	*p* value	*β* coefficient (95% CI)	*p* value
MoCA	-0.33 (-0.56 to -0.11)	0.004	-0.075 (-0.31 to 0.16)	0.52
CASI	-1.16 (-1.76 to -0.56)	<0.001	-0.70 (-1.22 to -0.18)	0.009

^∗^Adjusting for stepwise procedure selected covariates (age, sex, smoking habit, a history of diabetes, hypertension, coronary artery disease, and cerebrovascular disease, systolic and diastolic blood pressures, body mass index, log-transformed hemodialysis duration, cause of end-stage renal disease, albumin, log-transformed triglyceride, total cholesterol, hemoglobin, creatinine and calcium-phosphorus product, Kt/V, and amount of ultrafiltration).

## Data Availability

The data used to support the findings of this study are available from the corresponding author upon request.

## Supplementary Materials

Supplementary Materials Supplementary Table 1: description of the neuropsychiatric test on patients with hemodialysis in the study. Supplementary Table 2: determinants of MoCA and ABI association using multivariate stepwise linear regression analysis. Supplementary Table 3: determinants of CASI and ABI association using multivariate stepwise linear regression analysis. Supplementary Table 4: determinants of MoCA and baPWV association using multivariate stepwise linear regression analysis. Supplementary Table 5: determinants of CASI and baPWV association using multivariate stepwise linear regression analysis. Supplementary Table 6: the association between comorbidities and cognitive function (MoCA and CASI). Supplementary Table 7: the association between ABI and cognitive function test (MoCA and CASI) using multivariate stepwise linear regression analysis and further additional diabetes mellitus comorbidity adjustment. Supplementary Table 8: the association between baPWV and cognitive function test (MoCA and CASI) using multivariate stepwise linear regression analysis and further additional diabetes mellitus comorbidity adjustment.
