# Supplementary material for: A Low Ankle-Brachial Index and High Brachial-Ankle Pulse Wave Velocity Are Associated with Poor Cognitive Function in Patients Undergoing Hemodialysis
Source: Dis Markers. 2019 Aug 19;2019:9421352. doi: 10.1155/2019/9421352 (PMC6721107; doi:10.1155/2019/9421352)
Supplement: Supplementary Materials — Supplementary Table 1: description of the neuropsychiatric test on patients with hemodialysis in the study. Supplementary Table 2: determinants of MoCA and ABI association using multivariate stepwise linear regression analysis. Supplementary Table 3: determinants of CASI and ABI association using multivariate stepwise linear regression analysis. Supplementary Table 4: determinants of MoCA and baPWV association using multivariate stepwise linear regression analysis. Supplementary Table 5: determinants of CASI and baPWV association using multivariate stepwise linear regression analysis. Supplementary Table 6: the association between comorbidities and cognitive function (MoCA and CASI). Supplementary Table 7: the association between ABI and cognitive function test (MoCA and CASI) using multivariate stepwise linear regression analysis and further additional diabetes mellitus comorbidity adjustment. Supplementary Table 8: the association between baPWV and cognitive function test (MoCA and CASI) using multivariate stepwise linear regression analysis and further additional diabetes mellitus comorbidity adjustment. [file 9421352.f1.pdf]

Supplementary Table 1. Description of the neuropsychiatric test on patients with hemodialysis in the study

Neuropsychiatric test	Description	Definition of cognitive impairment
Montreal Cognitive Assessment (MoCA)	The MoCA is a brief screening instrument assessing several cognitive domains including attention, executive functions, language, memory, and orientation. It is available in several languages and is widely used to detect cognitive impairment in numerous neurological and non-neurological diseases.	The maximum score of 30 points
Cognitive Abilities Screening Instrument (CASI)	The CASI is a 40-item global cognitive test that assesses a broad range of cognitive domains. It has nine cognitive evaluation domain including long-term memory, short term memory, orientation, attention, mental manipulation, list-generating fluency, language, abstraction/judgment, and drawing.	Cognitive Abilities Screening Instrument, Chinese versions (CASI C-2.0)<br>The maximum score of 100 points and cut-off score based on different education levels (no formal education, received 1–5 years of schooling, received 6 or more years of education)

Supplementary Table 2. Determinants of MoCA and ABI association using multivariate stepwise linear regression analysis

Parameters	β coefficient (95% CI)	*p* value
ABI (per 0.1)	0.62 (0.14 to 1.09)	0.011
Age (per 1 year)	-0.27 (-0.35 to -0.18)	<0.001
Systolic blood pressure (per 1 mmHg)	-0.043 (-0.077 to -0.0083)	0.015
Sex (male *vs.* female)	2.12 (0.35 to 3.88)	0.019
Total cholesterol (per 1 mg/dL)	0.023 (0.0011 to 0.045)	0.040

Adjusting for stepwise procedure selected covariates (age, sex, smoking habit, a history of diabetes, hypertension, coronary artery disease and cerebrovascular disease, systolic and diastolic blood pressures, body mass index, log-transformed hemodialysis duration, cause of end-stage renal disease, albumin, log-transformed triglyceride, total cholesterol, hemoglobin, creatinine and calcium-phosphorus product, Kt/V, and amount of ultrafiltration)

Supplementary Table 3. Determinants of CASI and ABI association using multivariate stepwise linear regression analysis

Parameters	β coefficient (95% CI)	*p* value
ABI (per 0.1)	1.43 (0.17 to 2.70)	0.026
Age (per 1 year)	-0.74 (-0.96 to -0.52)	<0.001
Log transformed hemodialysis duration	5.60 (0.12 to 11.1)	0.045
Sex (male *vs.* female)	7.55 (2.82 to 12.3)	0.002
Total cholesterol (per 1 mg/dL)	0.080 (0.018 to 0.14)	0.012
Systolic blood pressure (per 1 mmHg)	-0.086 (-0.18 to 0.0051)	0.064
Log-transformed triglyceride	1.43 (0.17 to 2.70)	0.026

Adjusting for stepwise procedure selected covariates (age, sex, smoking habit, a history of diabetes, hypertension, coronary artery disease and cerebrovascular disease, systolic and diastolic blood pressures, body mass index, log-transformed hemodialysis duration, cause of end-stage renal disease, albumin, log-transformed triglyceride, total cholesterol, hemoglobin, creatinine and calcium-phosphorus product, Kt/V, and amount of ultrafiltration)

Supplementary Table 4. Determinants of MoCA and baPWV association using multivariate stepwise linear regression analysis

Parameters	β coefficient (95% CI)	*p* value
baPWV (per 100 cm/s)	-0.075 (-0.31 to 0.16)	0.520
Age (per 1 year)	-0.30 (-0.39 to -0.20)	<0.001
Sex (male *vs.* female)	2.04 (0.23 to 3.86)	0.027
Total cholesterol (per 1 mg/dL)	0.024 (0.0019 to 0.047)	0.034
Systolic blood pressure (per 1 mmHg)	-0.036 (-0.076 to 0.0049)	0.084
CaXP product (per 1 mg^2/dL^2)	-0.065 (-0.14 to 0.011)	0.091

Adjusting for stepwise procedure selected covariates (age, sex, smoking habit, a history of diabetes, hypertension, coronary artery disease and cerebrovascular disease, systolic and diastolic blood pressures, body mass index, log-transformed hemodialysis duration, cause of end-stage renal disease, albumin, log-transformed triglyceride, total cholesterol, hemoglobin, creatinine and calcium-phosphorus product, Kt/V, and amount of ultrafiltration)

Supplementary Table 5. Determinants of CASI and baPWV association using multivariate stepwise linear regression analysis

Parameters	β coefficient (95% CI)	*p* value
baPWV (per 100 cm/s)	-0.70 (-1.22 to -0.18)	0.009
Age (per 1 year)	-0.74 (-0.97 to -0.51)	<0.001
Sex (male *vs.* female)	7.00 (2.31 to 11.7)	0.004
Log transformed hemodialysis duration	6.52 (1.16 to 11.9)	0.017
Total cholesterol (per 1 mg/dL)	0.067 (0.0088 to 0.13)	0.024
CaXP product (per 1 mg^2/dL^2)	-0.21 (-0.41 to -0.016)	0.035

Adjusting for stepwise procedure selected covariates (age, sex, smoking habit, a history of diabetes, hypertension, coronary artery disease and cerebrovascular disease, systolic and diastolic blood pressures, body mass index, log-transformed hemodialysis duration, cause of end-stage renal disease, albumin, log-transformed triglyceride, total cholesterol, hemoglobin, creatinine and calcium-phosphorus product, Kt/V, and amount of ultrafiltration)

Supplementary Table 6. The association between comorbidities and cognitive function (MoCA and CASI)

	MoCA		CASI	
	β coefficient (95% CI)	*p* value	β coefficient (95% CI)	*p* value
Diabetes mellitus	-2.24 (-4.27 to -0.221)	0.03	-7.16 (-12.7 to -1.65)	0.011
Hypertension	0.431 (-1.62 to 2.48)	0.679	-0.91 (-6.54 to 4.72)	0.75
Stroke	-0.383 (-4.71 to 3.95)	0.861	-6.04 (-17.9 to 5.79)	0.315
Coronary artery disease	-2.73 (-6.29 to 0.835)	0.132	-9.17 (-18.9 to 0.548)	0.064

Supplementary Table 7. The association between ABI and cognitive function test (MoCA and CASI) using multivariate stepwise linear regression analysis and further additional diabetes mellitus comorbidity adjustment

Cognitive function test	Multivariate (stepwise)*		Multivariate (stepwise)* with DM adjustment	
	β coefficient (95% CI)	*p* value	β coefficient (95% CI)	*p* value
MoCA	0.62 (0.14 to 1.09)	0.011	0.564 (0.081 to 1.046)	0.022
CASI	1.43 (0.17 to 2.70)	0.026	1.363 (0.042 to 2.683)	0.043

* Adjusting for stepwise procedure selected covariates (age, sex, smoking habit, a history of diabetes, hypertension, coronary artery disease and cerebrovascular disease, systolic and diastolic blood pressures, body mass index, log-transformed hemodialysis duration, cause of end-stage renal disease, albumin, log-transformed triglyceride, total cholesterol, hemoglobin, creatinine and calcium-phosphorus product, Kt/V, and amount of ultrafiltration)

Supplementary Table 8. The association between baPWV and cognitive function test (MoCA and CASI) using multivariate stepwise linear regression analysis and further additional diabetes mellitus comorbidity adjustment

Cognitive function test	Multivariate (stepwise)*		Multivariate (stepwise)* with DM adjustment	
	β coefficient (95% CI)	*p* value	β coefficient (95% CI)	*p* value
MoCA	-0.075 (-0.31 to 0.16)	0.52	-0.06 (-0.293 to 0.166)	0.582
CASI	-0.70 (-1.22 to -0.18)	0.009	-0.661 (-1.191 to -0.131)	0.015

* Adjusting for stepwise procedure selected covariates (age, sex, smoking habit, a history of diabetes, hypertension, coronary artery disease and cerebrovascular disease, systolic and diastolic blood pressures, body mass index, log-transformed hemodialysis duration, cause of end-stage renal disease, albumin, log-transformed triglyceride, total cholesterol, hemoglobin, creatinine and calcium-phosphorus product, Kt/V, and amount of ultrafiltration)
